# Pain and removal force associated with bracket debonding: a clinical study

**DOI:** 10.1590/1678-7757-2020-0879

**Published:** 2021-07-23

**Authors:** Narumi Nakada, Yasuki Uchida, Mizuki Inaba, Ryo Kaetsu, Natsuo Shimizu, Yasuhiro Namura, Mitsuru Motoyoshi

**Affiliations:** 1 Nihon University School of Dentistry Department of Orthodontics Chiyoda-kuTokyo Japan Nihon University School of Dentistry, Department of Orthodontics, Chiyoda-ku, Tokyo, Japan.; 2 Nihon University School of Dentistry Division of Clinical Research Dental Research Center Chiyoda-kuTokyo Japan Nihon University School of Dentistry, Division of Clinical Research, Dental Research Center, Chiyoda-ku, Tokyo, Japan.; 3 Nihon University Graduate School of Dentistry Department of Oral Structural and Functional Biology Chiyoda-kuTokyo Japan Nihon University Graduate School of Dentistry, Department of Oral Structural and Functional Biology, Chiyoda-ku, Tokyo, Japan.

**Keywords:** Dental debonding, Orthodontic brackets, Bond force dental cement

## Abstract

**Objective:**

Pain is a problem during bracket removal, and more comfortable treatment is needed. This study examined the association of pain with the removal force required for ceramic brackets, compared with metal and plastic brackets, to determine which removal method resulted in less pain and discomfort.

**Methodology:**

81 subjects (mean age, 25.1 years; 25 males and 56 females) were enrolled, from whom 1,235 brackets (407 ceramic, 432 plastic, and 396 metal) were removed. Measured teeth were distinguished at six segments. Pain was measured with a visual analogue scale (VAS) during the removal of each bracket. An additional grip was placed on the grips of debonding pliers with right-angled beaks; a mini loading cell sensor pinched by the grips was used to measure removal force during debonding. VAS and force values were statistically analyzed. The Kruskal–Wallis test followed by the Mann–Whitney U test with Bonferroni correction were performed for multiple comparisons; multiple regression analysis was also performed.

**Results:**

Forces in the upper and lower anterior segments were significantly smaller (p<0.05) than those in the other segments. Pain tended to be greater in the upper and lower anterior segments than in the posterior segments. In all segments, the removal force was greater for metal brackets than for plastic or ceramic brackets. Ceramic brackets caused significantly greater pain than plastic brackets for the upper and lower anterior segments. Debonding force was involved in the brackets, following adjustments for pain, upper left segment, age, and sex.

**Conclusions:**

Pain and discomfort are likely to occur during bracket debonding.

## Introduction

Ceramic brackets have been used as an aesthetic option in treatment of both adults and adolescents. Although ceramic brackets are aesthetically superior to other types, they are more difficult to remove by orthodontists and cause more discomfort for patients in the clinic. The increased fracture incidence and complicated debonding process of alumina ceramic brackets are problematic because brackets are mechanically strong but brittle.^1^^,^^2^ Alexopoulou, et al.^3^ (2020) reported that the mechanical properties of alumina brackets are inferior after intraoral exposure. Single-crystal and polycrystalline brackets share an equal mechanical Martens hardness and elastic modulus, but the single-crystal type is more brittle.^3^

Many researchers have studied pain during bracket removal.^4^^-^^9^ Williams and Bishara^10^ (1992) conducted a pilot study of 15 patients to investigate the level and direction of force that could be tolerated during debonding. Torsional forces were very poorly tolerated, and less than 100 g of force could be applied before discomfort was experienced; intrusion forces were relatively well tolerated, with a discomfort threshold of 934 g. Teeth mobility reduced the discomfort threshold. Mangnall, et al.^11^ (2013) investigated the effect of a pain score during debonding with/without a wafer. They observed a relationship between pain and the debonding segment; moreover, they found that pain in the upper right posterior segment was relatively high because it was the first segment removed and the operator had to rotate their hand position.

To the best of our knowledge, few quantitative studies have been performed concerning the relationship between discomfort and ceramic bracket removal force. In addition, it is unknown whether the pain threshold decreases after the first segment is removed. This study examined the associations of pain with removal forces of ceramic, metal, and plastic brackets. It also tested a removal method with less pain and discomfort. The hypothesis is that there is no difference in pain perception/discomfort or removal force among brackets.

## Methodology

Ethical approval (EP-18D004) was obtained from the Nihon University School of Dentistry Ethics Committee. The sample size (≥289 brackets) was calculated by setting a 95% confidence interval and 80% power value, using the mean and standard deviation discomfort threshold values (900.0±184.1 grams in males and 855.0±201.1 grams in females) during debonding reported by Williams and Bishara^10^ (1992). Eighty-one patients (25 males aged 25.8±9.3 years, 422 brackets; and 56 females aged 24.7±8.5 years, 813 brackets) were selected (Tables 1 and 2). In many patients, different brackets (e.g., ceramic brackets on anterior teeth and metal brackets on premolars were worn) were used for the same patient. The inclusion criteria were the need for bracket removal and provision of informed consent, wearing brackets bonded with the adhesives described below, and good general health. The exclusion criteria were absence of caries, fillings, periodontal disease, hypoplasia in bracket-removed surfaces, medical history of chronic diseases, and chronic self-medication, especially with anxiolytic drugs.

**Table 1:** Subjects number, age and treatment duration

	Subjects (number)	Brackets (number)	Age (years)		Treatment duration (months)	
			Mean	SD	Mean	SD
Male	25	422	25.8	9.3	45.0	16.0
Female	56	813	24.7	8.5	39.7	20.7
Total	81	1235	25.1	8.7	41.3	19.6

**Table 2:** Detail of brackets

	Ceramic	Plastic	Metal	Total
Number	407	432	396	1235
Total number of subjects	43	50	70	163

**Figure 1:**
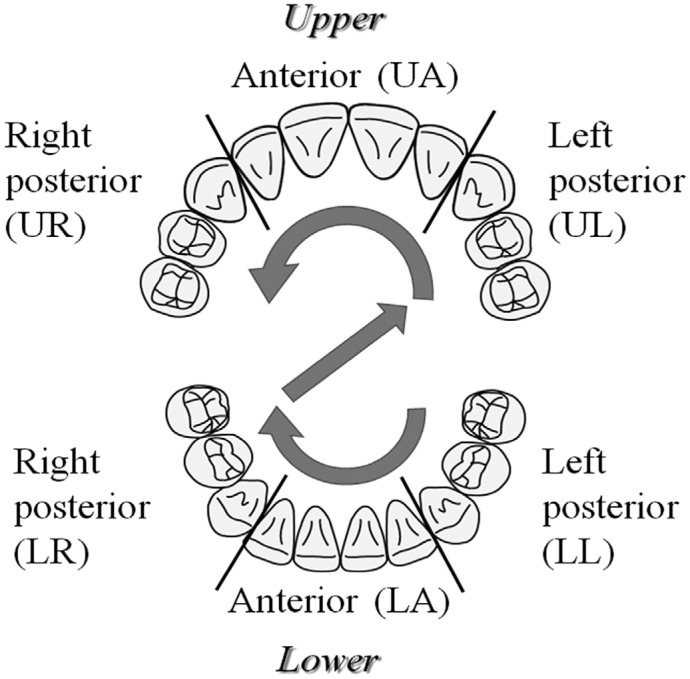
Allocation of the teeth. Arrows indicate the order of bracket removal

**Figure 2:**
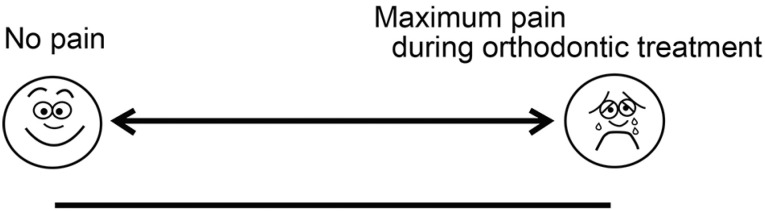
The visual analogue scale (VAS) used in this study. Patients drew a perpendicular line somewhere along the VAS according to the intensity of the pain felt

The measured teeth were distinguished into six segments (Figure 1): the anterior segments included the central and lateral incisors, while the posterior segments included the canines, first premolars, and second premolars. The brackets were first removed from the lower left posterior segment, followed by the lower anterior (LA), lower right posterior, upper left posterior, upper anterior (UA), and upper right posterior segments, in that order. An operator instructed the patients to draw a perpendicular line on a visual analogue scale (VAS) to denote the intensity of pain after each bracket was removed. The VAS consisted of a 100-mm line labelled at the extremes with ‘no pain’ and a happy face and ‘maximum pain during orthodontic treatment’ and a sad face (Figure 2). The distance from the zero point to the perpendicular line was measured and taken to indicate pain severity. All removal force and VAS measurements were obtained by one operator (N.N).

**Figure 3:**
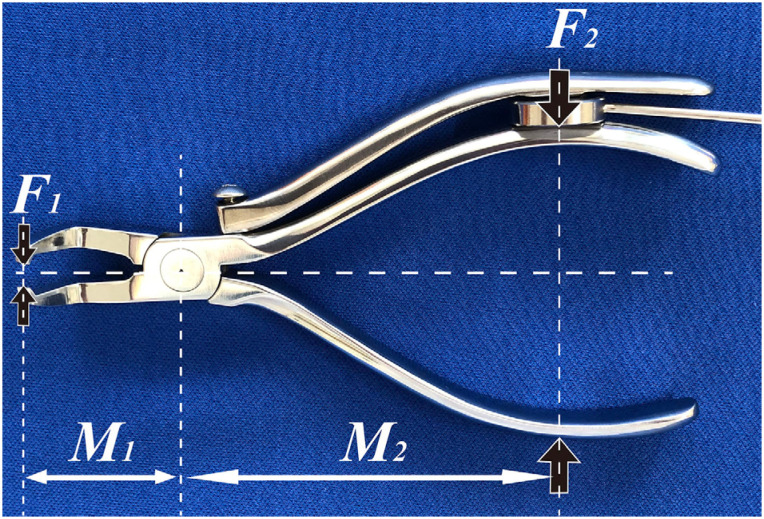
Debonding pliers. A sensor was located between the grip of the pliers and the additional grip

Some studies have reported removal forces determined *in vivo* using various bracket removal tools.^12^^-^^15^ In the present study, a modified right-angled beak bracket-removing plier, which has an additional grip, was used for measuring the removal forces. A mini loading cell sensor pinched by the grips measured the force squeezing the grips during debonding (Figure 3). The squeezing force applied to the plier, which pushes the sensor, is translated by the bracket removal force, *F_1_*, and the measured squeezing force, *F_2_*, is given as follows:^16^

$$F_{1}M_{1}=F_{2}M_{2}$$

Then,

$$F_{1}=F_{2}M_{2}/M_{1}$$

Where *M_1_* and *M_2_* are the moment arms in Figure 3.

Bracket removal force was compared between the force obtained from the debonding pliers with an attached sensor and the force obtained from a universal testing machine (as a pilot test) before measuring the samples. Briefly, 16 metal brackets (mesh brackets; Tomy International, Tokyo, Japan) were bonded to bovine teeth with a light-curing bonding agent (Light Bond sealant with fluoride, Reliance Orthodontic Products, Itasca, IL, USA) followed by adhesive paste (Transbond XT light cure adhesive paste, 3M Unitek, Minneapolis, MN, USA), and the specimens were stored in distilled water at 37°C. After 24 hours, the debonding force of each of the eight bonded brackets was measured with the debonding pliers with an attached sensor and the universal testing machine under the shear mode condition with a crosshead speed of 1 mm/min.

During the standardized *in vivo* debonding procedure, the teeth were adjunctively seated in the direction of the tooth axis during bracket removal because mobility of the teeth reduces the discomfort threshold;^17^ the tip on the gingival side of the plier was used to hold the bracket base, and the tooth crown was held with cotton wool and the operator's fingers to fix it in the axial direction as the grips of the plier were squeezed for bracket removal. The brackets removed from the teeth were of three types: ceramic (InVu, TP Orthodontics, Warsaw, IN, USA), plastic (ortho Esta MB, Tomy International), and metal (Mesh brackets, Tomy International). All brackets (total of 1,235 brackets; 407 ceramic, 432 plastic, and 396 metal) were bonded with a light-curing bonding agent (Light Bond sealant with fluoride) and adhesive paste (Light Bond light cure adhesive paste or Transbond XT light cure adhesive paste).

### Statistical analysis

The VAS and force value results were analysed with IBM SPSS Statistics, version 23 (IBM Corp., Armonk, NY, USA). The Kolmogorov–Smirnov and Levene tests were used to verify the normality and homogeneity of the variance. The t-test was used to compare removal forces from the pilot test. The Kruskal–Wallis test followed by the Mann–Whitney U test with Bonferroni correction was performed for multiple comparisons because the VAS results were not normally distributed. Multiple regression analysis was performed after logarithmic transformation of the VAS results. P-values < 0.05 were considered statistically significant.

## Results

### Pilot test removal force

Mean removal forces ± standard deviations were 146.54±42.56 N with the universal testing machine and 149.05±54.32 N with the debonding pliers (F-value=0.229, effect size r=0.020, d=0.031).

### Multiple comparisons

**Figure 4:**
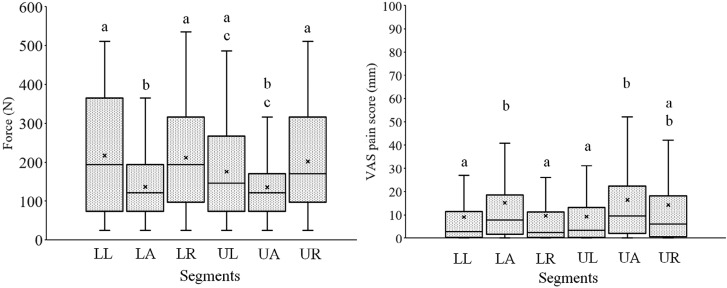
Box-and-whisker plots of removal forces and pain for each segment. In boxes with bars, maximum, first quartile, median, third quartile, and minimum values are indicated in order from the top. Cross mark indicates average value. Different letters between segments indicate a significant difference (p<0.05). Forces were significantly smaller on upper and lower anterior segments than on other segments

**Figure 5:**
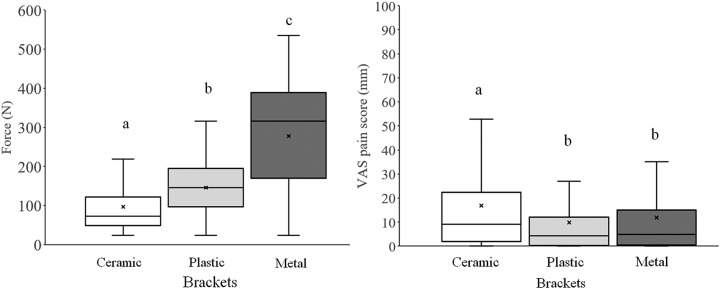
Box-and-whisker plots of removal forces and pain for each bracket. The elements of the box plot are identical to the elements in Figure 4. Different letters between bracket groups indicate a significant difference (p<0.05). The debonding force was significantly higher for the metal bracket than for other brackets

Before applying the Bonferroni multiple comparison test, removal forces and pain were compared between the sexes using the Mann–Whitney *U* test. No significant difference was observed in force or pain (males: 171.4±121.7 N and 11.5±15.0 mm; females: 172.4±119.1 N and 13.5±19.8 mm, respectively). Removal forces and pain during debonding among the segments are shown in Figure 4. Forces were significantly smaller in the UA and LA segments than in other segments. Pain tended to be greater in the UA and LA segments than in the posterior segments. The forces, pain, and types of brackets are shown in Figure 5. The removal force was the greatest for metal brackets, followed by plastic and ceramic brackets; pain from ceramic brackets was the highest, compared to the other brackets.

Forces and pain according to the types of brackets in each segment are shown in Table 3. In all segments, the removal forces of metal brackets were higher than the removal forces of plastic and ceramic brackets. Pain in the LA and UA segments was significantly greater for ceramic brackets than for plastic brackets. The pain was not dependent on bracket type in any of the posterior segments. Force and pain data in the LA and UA segments are shown in Table 4. No significant difference was observed between the incisors, in the force or VAS pain score, in any segment.

**Table 3:** Force (N) and VAS score in each segment

	Force (N)												VAS (mm)											
	Ceramic				Plastic				Metal				Ceramic				Plastic				Metal			
Segment	Median	Mean	SD	group*	Median	Mean	SD	group*	Median	Mean	SD	group*	Median	Mean	SD	group*	Median	Mean	SD	group*	Median	Mean	SD	group*
LL	72.9	77.8	41.8	a	145.9	144.3	74.4	b	328.3	293	141.6	c	7.9	13.3	16.2	a	0.9	5.7	10.3	a	3.1	9.3	13.8	a
LA	72.9	96.3	65.8	a	145.9	146.5	75.4	b	316.1	285.7	136.6	c	12.6	20.8	21.1	a	6.9	12.2	19.6	b	8.3	7.6	6.7	a,b
LR	85.1	99.7	54.5	a	121.6	135.0	78.7	a	291.8	278.8	125.1	b	2.2	7.3	12.0	a	1.3	6.6	10.2	a	3.1	11.6	17.8	a
UL	72.9	86.6	64.1	a	133.7	133.0	58.7	b	243.1	247.1	140.9	c	3.9	8.7	12.7	a	1.4	7.3	13.3	a	4.2	10.4	17.4	a
UA	97.3	100.8	54.9	a	145.9	148.7	57.4	b	364.7	373.8	78.3	c	10.1	20.1	22.0	a	5.5	11.0	15.0	b	12.1	16.2	14.3	a,b
UR	85.1	102.8	59.8	a	170.2	165.9	63.1	b	303.9	268.9	128.1	c	6.0	13.8	20.3	a	1.4	8.0	11.9	a	6.6	16.9	23.1	a

* Different letters in forces and VAS in each segment indicate a significant difference between brackets (p < 0.05).

SD: standard deviation, LL: lower left, LA: lower anterior, LR: lower right, UL: upper left, UA: upper anterior, UR: upper right.

**Table 4:** Force (N) and VAS score in incisors

	Force (N)			VAS (mm)		
	Median	Mean	SD	Median	Mean	SD
Lower						
Left lateral incisor	121.6	127.0	74.5	6.83	12.5	17.4
Left central incisor	121.6	133.2	93.6	8.8	15.6	18.5
Right central incisor	121.6	136.8	88.4	9.45	18.1	22.7
Right lateral incisor	121.6	148.6	98.2	7.71	14.2	21.7
Upper						
Left lateral incisor	121.6	128.1	81.4	9.59	17.7	20.1
Left central incisor	145.9	146.2	86.8	9.93	16.9	20.6
Right central incisor	121.6	132.4	87.2	7.04	15.3	19.4
Right lateral incisor	121.6	132.5	85.4	7.97	15.4	19.0

SD: Standard deviation.

No significant differences were detected between the incisors for force and VAS respectively.

### Multiple regression analysis

We performed multiple regression analysis and interpreted the force factor as a dependent variable. The correlation coefficient (Table 5) was 0.653, and this result was indicated by predictors of (constant), metal bracket, ceramic bracket, UL segment, logarithmic VAS, sex, and age. Simultaneously, a significant difference (p<0.05) in analysis of variance was observed. The variance inflation factor values were small, and no collinearity was observed (Table 6). Therefore, the debonding force was predicted by the bracket type, upper left posterior segment, logarithmic VAS, sex, and age.

**Table 5:** Model summary

Model	R	R^2^	Adjusted R^2^	Standard error of the estimate
1	0.612^a^	0.375	0.374	93.969
2	0.636^b^	0.405	0.404	91.743
3	0.643^c^	0.413	0.411	91.153
4	0.648^d^	0.420	0.418	90.664
5	0.650^e^	0.423	0.420	90.467
6	0.653^f^	0.427	0.423	90.214

a Predictors: (constant), metal bracket.

b Predictors: (constant), metal bracket, ceramic bracket.

c Predictors: (constant), metal bracket, ceramic bracket, UL segement.

d Predictors: (constant), metal bracket, ceramic bracket, UL segement, logarithmic VAS.

e Predictors: (constant), metal bracket, ceramic bracket, UL segement, logarithmic VAS, sex.

f Predictors: (constant), metal bracket, ceramic bracket, UL segement, logarithmic VAS, sex, age.

**Table 6:** Coefficient

	Unstandardized coefficient		Standardized coefficient			Collinearity statistics	
model	B	Standard error	beta	t	P value	Tolerance	VIF
(constant)	122.234	10.662		11.464	0		
metal	136.71	7.124	0.537	19.19	0	0.719	1.39
ceramic	−51.943	6.979	−0.208	−7.442	0	0.719	1.39
UL segment	−30.833	8.675	−0.086	−3.554	0	0.972	1.029
logarithmic VAS	6.981	1.836	0.092	3.803	0	0.956	1.046
sex	−16.543	6.039	−0.067	−2.74	0.006	0.95	1.052
age	0.898	0.347	0.063	2.591	0.01	0.956	1.046

VIF:Variance inflation factor

## Discussion

Removal forces significantly differed among the brackets used in this study. Thus, the hypothesis made before conducting the study was rejected. The base of the ceramic brackets used in this study was composed of a thick layer of plastic.^18^ Generally, bond fracture occurs where the bonding layer is grasped with the tip of the remover. Therefore, stress is applied to the adhesive itself, as well as the interface between the adhesive and the brackets or teeth. The base of the ceramic brackets used in this study was more easily grasped with the tip of the removal plier. As a result, the removal forces acting on the ceramic brackets were the smallest among all brackets used. However, pain was greatest during removal of ceramic brackets. This may be due to the impact that occurs during the removal of the bracket, in addition to the way in which the bracket is grasped. Using metal or plastic brackets, it is difficult to grasp the thin bracket base or adhesive layer, and the bracket body continues to be squeezed until bond destruction occurs. In the case of ceramic brackets, removal force is applied to the bracket base instantaneously. Therefore, although the impact during removal varies depending on material properties, such as stiffness, the impact during removal of the ceramic brackets used in this study, which had a harder plastic base than the plastic brackets, was presumably the source of the greater discomfort.

Moreover, the pain that patients experienced during orthodontic treatment was set as the maximum VAS value during debonding in this study; thus, most VAS values during removal were less than 25% of the maximum pain during orthodontic treatment and it was found the pain of removal was considerably lower than the pain that patients experienced during orthodontic treatment. In a previous study^19^ comparing orthodontic aligners and fixed appliances, pain intensity was similar for all time points at which pain was recorded. In another previous study investigating the pain caused by orthodontic appliances, a mean VAS pain score of ∼40% was reported, and pain reached a peak after a few days.^20^^,^^21^ According to these findings, pain perception during bracket removal is mild and transient, which is clinically irrelevant.

Also, we did not distinguish between pain and discomfort, because we considered the subtle difference between those constructs would not be detected with the VAS. Some patients may not perceive pain during debonding. In the case of mild pain, perception thereof may be “converted” by other factors, such as anxiety, into discomfort. It should also be noted gender, age, and daily life activities are considered to affect perceptions of pain and discomfort.^22^ Physical complaints and psychological factors, such as cognition, socialization, and personality, have also been reported to be affected by increased pain.^23^ Mendonca, et al.^21^ (2020) reported that anxious patients had higher pain levels during the initial phase of orthodontic treatment. Thus, anxiety is a key aspect of pain perception, and any kind of communication that eliminates anxiety could also affect pain perception during removal of brackets.

In this study, the force during debonding was expressed indirectly as the force detected by a sensor on the grip of the pliers. The actual shearing force of the bracket was calculated from the distances between the fulcrum and the points of effort and load. Horiuchi, et al.^16^ (2009) reported a comparison between orthodontic adhesives by converting force at the plier grips to bond strength. No significant differences in removal force were observed between the universal testing machine and the pliers during the comparative pilot test in our study. The data distributions of each group overlapped broadly because of extremely small effect sizes. Therefore, the force detected at the grips was converted to bond force even if the values may be identical.

Bond strengths of orthodontic adhesives have been reported to range from 3.96 to 20.1 MPa *in vivo*^24^ and from 3.50 to 27.76 MPa *in vitro*.^25^ In this study, when the debonding force was converted using the bracket base area and distances between the fulcrum and the points of effort and load, the mean bond strengths ranged from 5.28 to 9.00 MPa. Considering the adhesive aging,^26^ the bond strength of the ceramic brackets (InVu brackets) was similar to the results in a previous study in which the same ceramic brackets were used.^18^^,^ ^27^ The tip of the removing pliers held the bracket body, rather than the thin bracket base or adhesive layer, when the metal and plastic brackets were debonded. Therefore, the debonding force presumably exhibited greater enhancement for the ceramic bracket.

The first removing segment was not the most painful in this study (Figure 4). Although bracket removal began with the lower left posterior segment in all patients, greater pain tended to be experienced at the UA and LA segments. Mangnall, et al.^11^ (2013) reported the initiation of debonding at the upper right posterior segment; the pain value in that segment was 18%. The lowest pain value was 6% in both the upper and lower posterior segments, while the greatest pain value was 39% in the LA segment. In our study, pain was higher in the anterior segment, although the debonding force was lower.

The predicted debonding force was derived from multiple regression analysis. The results showed that, among potential predictive factors (bracket type, pain, upper left posterior segment, age, and sex), the main predictive factor was the bracket type, for which the coefficient was greatest. Holding the bracket-removing pliers with right-angled beaks to the left side of the segment was easier than holding those pliers to the right side of the segment. This is presumably because the debonding force was more appropriate in the upper left segment than in other segments.

In a study involving multiple regression analyses, Mangnall, et al.^11^ (2013) reported that the wafer group had significantly less pain during debonding of the upper posterior and lower posterior teeth. Debonding with a wafer prevented impact; debonding with a similarly effective, intrusive, stabilising force could also be achieved by using a cotton wool roll.^11^ Williams and Bishara^10^ (1992) stated that the type of force applied affects the threshold for discomfort: intrusive forces were tolerated best, while torsional forces were poorly tolerated. In our study, it was difficult to use debonding pliers with a wafer holding the lower anterior teeth, because the tip of the pliers struck the gingiva and held the wafer. Therefore, stabilisation during debonding was achieved by holding a small amount of cotton wool between the orthodontist's fingers.

This study found removal of plastic brackets was associated with less discomfort than removal of ceramic brackets. However, considering the poorer quality of plastic brackets, the comparison may not be of clinical significance. Debonding brackets may induce to only mild pain and considering that this sensation is temporary as described above, the decision to use a specific type of bracket should rely on required mechanics and patients’ expectation regarding aesthetics and costs rather than in pain perception for removal. Thus, the differences in pain between ceramic and plastic brackets debonding may not be clinically significant.

Reports of significant differences in pain coexist with others reporting no significant differences.^22^ Regarding age, although it is not possible to compare studies because of differences in experimental designs, Brown and Moerenhout^28^ (1991) reported adolescents were more vulnerable to the undesirable psychological effects of treatment and had higher levels of pain than both younger and older patients. Scheurer, et al.^22^ (1996) similarly stated their middle age group (13–16 years) had the highest pain frequency during orthodontic therapy. Moreover, the types of brackets compared has been limited, and a limitation of this study was that sex and age group differences in tooth substances and bracket types were not investigated.

## Conclusion

Within the limitations of the current study, the following conclusions can be drawn:

Pain was not associated with debonding order and was not dependent on bracket type in any posterior segment.

Forces were significantly smaller in the upper and lower anterior segments than in other segments. Pain was greater in the upper and lower anterior segments than in the posterior segments.

Ceramic brackets required less removal force than plastic brackets, but greater pain was detected in the upper and lower incisors.

## References

[1] 1- Bishara SE, Fehr DE. Ceramic brackets: something old, something new, a review. Semin in Orthod. 1997;3(3):178-88. doi: 10.1016/s1073-8746(97)80068-0 10.1016/s1073-8746(97)80068-0 9573879

[2] 2- Ryu C, Namura Y, Tsuruoka T, Hama T, Kaji K, Shimizu N. The use of easily debondable orthodontic adhesives with ceramic brackets. Dent Mater J. 2011;30(5):642-7. doi: 10.4012/dmj.2011-013 10.4012/dmj.2011-013 21946484

[3] 3- Alexopoulou E, Polychronis G, Konstantonis D, Sifakakis I, Zinelis S, Eliades T. A study of the mechanical properties of as-received and intraorally exposed single-crystal and polycrystalline orthodontic ceramic brackets. Eur J Orthod. 2020;42(1):72-7. doi: 10.1093/ejo/cjz024 10.1093/ejo/cjz024 31009950

[4] 4- Kraut J, Radin S, Trowbridge HI, Emling RC, Yankell SL. Clinical evaluations on thermal versus mechanical debonding of ceramic brackets. J Clin Dent. 1991;2(4):92-6 1812905

[5] 5- Normando TS, Calçada FS, Ursi WJ, Normando D. Patients’ report of discomfort and pain during debonding of orthodontic brackets: a comparative study of two methods. World J Orthod. 2010;11(4):e29-34 21490985

[6] 6- Khan H, Chaudhry AR, Ahmad F, Warriach F. Comparison of debonding time and pain between three different debonding techniques for stainless steel brackets. Pak Oral Dent J. 2015;35(1):79-82

[7] 7- Pithon MM, Santos Fonseca Figueiredo D, Oliveira DD, Coqueiro RS. What is the best method for debonding metallic brackets from the patient's perspective? Prog Orthod. 2015;16:17. doi: 10.1186/s40510-015-0088-7 10.1186/s40510-015-0088-7 PMC4469684 26081783

[8] 8- Bavbek NC, Tuncer BB, Tortop T, Celik B. Efficacy of different methods to reduce pain during debonding of orthodontic brackets. Angle Orthod. 2016;86(6):917-24. doi: 10.2319/020116-88R.1 10.2319/020116-88R.1 PMC8597343 27172508

[9] 9- Priya A, Jain RK, Santhosh Kumar MP. Efficacy of different methods to reduce pain during debonding of orthodontic brackets. Drug Invention Today. 2018;10(9):1700-3

[10] 10- Williams OL, Bishara SE. Patient discomfort levels at the time of debonding: a pilot study. Am J Orthod Dentofacial Orthop. 1992;101(4):313-7. doi: 10.1016/S0889-5406(05)80324-5 10.1016/S0889-5406(05)80324-5 1558060

[11] 11- Mangnall LA, Dietrich T, Scholey JM. A randomized controlled trial to assess the pain associated with the debond of orthodontic fixed appliances. J Orthod. 2013;40(3):188-96. doi: 10.1179/1465313313Y.0000000045 10.1179/1465313313Y.0000000045 PMC4161196 24009318

[12] 12- Hajrassie MK, Khier SE. *In-vivo* and *in-vitro* comparison of bond strengths of orthodontic brackets bonded to enamel and debonded at various times. Am J Orthod Dentofacial Orthop. 2007;131(3):384-90. doi: 10.1016/j.ajodo.2005.06.025 10.1016/j.ajodo.2005.06.025 17346595

[13] 13- Hildebrand NK, Raboud DW, Heo G, Nelson AE, Major PW. Argon laser vs conventional visible light-cured orthodontic bracket bonding: an *in-vivo* and *in-vitro* study. Am J Orthod Dentofacial Orthop. 2007;131(4):530-6. doi: 10.1016/j.ajodo.2005.06.029 10.1016/j.ajodo.2005.06.029 17418721

[14] 14- Prietsch JR, Spohr AM, Lima da Silva IN, Pinheiro Beck JC, Silva Oshima HM. Development of a device to measure bracket debonding force *in vivo*. Eur J Orthod. 2007;29(6):564-70. doi: 10.1093/ejo/cjm069 10.1093/ejo/cjm069 17804426

[15] 15- Ahmed T, Rahman NA, Alam MK. Validation and reliability of a prototype orthodontic bracket debonding device equipped with force-sensitive resistor (FSR): a novel method of measuring orthodontic bracket debonding force *in vivo*. Prog Orthod. 2019;20(1):26. doi: 10.1186/s40510-019-0277-x 10.1186/s40510-019-0277-x PMC6612523 31281954

[16] 16- Horiuchi S, Kaneko K, Mori H, Kawakami E, Tsukahara T, Yamamoto K, et al. Enamel bonding of self-etching and phosphoric acid-etching orthodontic adhesives in simulated clinical conditions: debonding force and enamel surface. Dent Mater J. 2009;28(4):419-25. doi: 10.4012/dmj.28.419 10.4012/dmj.28.419 19721278

[17] 17- Almuzian M, Rizk MZ, Ulhaq A, Alharbi F, Alomari S, Mohammed H. Effectiveness of different debonding techniques and adjunctive methods on pain and discomfort perception during debonding fixed orthodontic appliances: a systematic review. Eur J Orthod. 2019;41(5): 486-94. doi: 10.1093/ejo/cjz013 10.1093/ejo/cjz013 30934051

[18] 18- Elekdag-Turk S, Isci D, Ozkalayci N, Turk T. Debonding characteristics of a polymer mesh base ceramic bracket bonded with two different conditioning methods. Eur J Orthod. 2009;31(1):84-9. doi: 10.1093/ejo/cjn067 10.1093/ejo/cjn067 19164413

[19] 19- Casteluci CE, Oltramari PV, Conti PC, Bonjardim LR, Almeida-Pedrin RR, Fernandes TMF, et al. Evaluation of pain intensity in patients treated with aligners and conventional fixed appliances: randomized clinical trial. Orthod Craniofac Res. 2020 Oct 15. doi: 10.1111/ocr.12431 10.1111/ocr.12431 33058419

[20] 20- Ireland AJ, Ellis P, Jordan A, Bradley R, Ewings P, Atack NE, et al. Comparative assessment of chewing gum and ibuprofen in the management of orthodontic pain with fixed appliances: a pragmatic multicenter randomized controlled trial. Am J Orthod Dentofacial Orthop. 2016;150(2):220-7. doi: 10.1016/j.ajodo.2016.02.018 10.1016/j.ajodo.2016.02.018 27476354

[21] 21- Mendonça DL, Almeida-Pedrin RR, Pereira NC, Oltramari PVP, Fernandes TM, Conti AC. The influence of text messages and anxiety on pain perception and its impact on orthodontic patients routine. Dental Press J Orthod. 2020;25(5):30-37. doi: 10.1590/2177-6709.25.5.030-037.oar 10.1590/2177-6709.25.5.030-037.oar PMC7668061 33206826

[22] 22- Scheurer PA, Firestone AR, Bürgin WB. Perception of pain as a result of orthodontic treatment with fixed appliances. Eur J Orthod. 1996;18(4):349-57. doi: 10.1093/ejo/18.4.349 10.1093/ejo/18.4.349 8921656

[23] 23- Jones M, Chan C. The pain and discomfort experienced during orthodontic treatment: a randomized controlled clinical trial of two initial aligning arch wires. Am J Orthod Dentofacial Orthop. 1992;102(4):373-81. doi: 10.1016/0889-5406(92)70054-e 10.1016/0889-5406(92)70054-e 1456222

[24] 24- Ahmed T, Rahman NA, Alam MK. Assessment of *in vivo* bond strength studies of the orthodontic bracket-adhesive system: a systematic review. Eur J Dent. 2018;12(4):602-9. doi: 10.4103/ejd.ejd_22_18 10.4103/ejd.ejd_22_18 PMC6178669 30369810

[25] 25- Finnema KJ, Ozcan M, Post WJ, Ren Y, Dijkstra PU. *In-vitro* orthodontic bond strength testing: a systematic review and meta-analysis. Am J Orthod Dentofacial Orthop. 2010;137(5):615-22.e3. doi: 10.1016/j.ajodo.2009.12.021 10.1016/j.ajodo.2009.12.021 20451780

[26] 26- Oesterlea LJ, Shellhartb WC. Effect of aging on the shear bond strength of orthodontic brackets. Am J Orthod Dentofacial Orthop. 2008;133(5):716-20. doi: 10.1016/j.ajodo.2006.04.042 10.1016/j.ajodo.2006.04.042 18456145

[27] 27- Rocha JM, Gravina MA, Silva Campos MJ, Quintão CC, Elias CN, Vitral RW. Shear bond resistance and enamel surface comparison after the bonding and debonding of ceramic and metallic brackets. Dental Press J Orthod. 2014;19(1):77-85. doi: 10.1590/2176-9451.19.1.077-085.oar 10.1590/2176-9451.19.1.077-085.oar PMC4299420 24713563

[28] 28- Brown DF, Moerenhout RG. The pain experience and psychological adjustment to orthodontic treatment of preadolescents, adolescents, and adults. Am J Orthod Dentofacial Orthop. 1991;100(4):349-56. doi: 10.1016/0889-5406(91)70073-6 10.1016/0889-5406(91)70073-6 1927986

